# (*E*)-*N*-{(*E*)-2-[(3,5-Dimethylbiphenyl-4-yl)imino]­acenaphthen-1-yl­idene}-2,6-di­methyl-4-phenyl­aniline

**DOI:** 10.1107/S1600536811054092

**Published:** 2011-12-21

**Authors:** Jianchao Yuan, Xiaoli Xie, Yufeng Liu, Chengping Miao, Jing Li

**Affiliations:** a Key Laboratory of Eco-Environment-Related Polymer Materials of the Ministry of Education, Key Laboratory of Polymer Materials of Gansu Province, College of Chemistry & Chemical Engineering, Northwest Normal University, Lanzhou 730070, People’s Republic of China

## Abstract

The title compound, C_40_H_32_N_2_, has crystallographic twofold rotation symmetry, with two C atoms lying on the axis. The dihedral angle between the two benzene rings of the 4-phenyl-2,6-dimethyl­phenyl group is 35.74 (17)°. The acenaphthene ring makes an angle of 76.93 (11)° with the benzene ring bonded to the N atom and an angle of 41.53 (13)° with the other benzene ring.

## Related literature

The title compound was synthesized as an α-diimine ligand for use in Ni^II^–α-diimine olefin polymerization catalysts. For applications of metal-organic polymerization catalysts, see: Johnson *et al.* (1995 ▶); Killian *et al.* (1996 ▶); Popeney *et al.* (2005 ▶, 2010 ▶, 2011 ▶). For a related structure, see: Lohr *et al.* (2011 ▶).
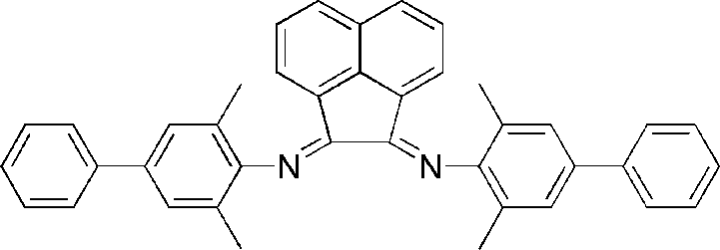


## Experimental

### 

#### Crystal data

C_40_H_32_N_2_
*M* *_r_* = 540.68
Monoclinic, 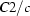

*a* = 22.994 (14) Å
*b* = 8.676 (5) Å
*c* = 18.652 (18) Å
β = 124.084 (4)°
*V* = 3082 (4) Å^3^
*Z* = 4
Mo *K*α radiation
μ = 0.07 mm^−1^
*T* = 296 K
0.23 × 0.21 × 0.19 mm

#### Data collection

Bruker APEXII CCD diffractometer
Absorption correction: multi-scan (*SADABS*; Sheldrick, 2008*a* ▶) *T*_min_ = 0.985, *T*_max_ = 0.987
10667 measured reflections
2857 independent reflections
1596 reflections with *I* > 2σ(*I*)
*R*_int_ = 0.031

#### Refinement

*R*[*F* ^2^ > 2σ(*F* ^2^)] = 0.059
*wR*(*F* ^2^) = 0.211
*S* = 1.16
2857 reflections
193 parameters
H-atom parameters constrained
Δρ_max_ = 0.23 e Å^−3^
Δρ_min_ = −0.20 e Å^−3^

### 

Data collection: *APEX2* (Bruker, 2008 ▶); cell refinement: *SAINT* (Bruker, 2008 ▶); data reduction: *SAINT*; program(s) used to solve structure: *SHELXS97* (Sheldrick, 2008*b* ▶); program(s) used to refine structure: *SHELXL97* (Sheldrick, 2008*b* ▶); molecular graphics: *SHELXTL* (Sheldrick, 2008*b* ▶); software used to prepare material for publication: *SHELXTL*.

## Supplementary Material

Crystal structure: contains datablock(s) I, global. DOI: 10.1107/S1600536811054092/fy2034sup1.cif

Structure factors: contains datablock(s) I. DOI: 10.1107/S1600536811054092/fy2034Isup2.hkl

Supplementary material file. DOI: 10.1107/S1600536811054092/fy2034Isup3.cml

Additional supplementary materials:  crystallographic information; 3D view; checkCIF report

## Figures and Tables

**Fig. 1.**
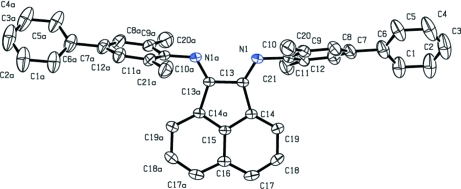
Structure of the title compound, with displacement ellipsoids drawn at the 30% probability level. H-atoms have been excluded for clarity. The atom name suffix indicates symmetry code (a): –x, y, –z + 1/2.

## supplementary crystallographic information

### Comment

In recent years, α-diimine nickel catalysts have drawn wide-spread attention due to their high catalytic activity in ethylene polymerization (Johnson *et al.*, 1995; Killian *et al.*, 1996). It is well known that the ligand structure has significant influence on the product properties and polymerization activities (Popeney *et al.*, 2011; Popeney *et al*., 2010; Popeney *et al.*, 2005). In this study, we designed and synthesized the title compound as a bidentate ligand, and its molecular structure was characterized by X-ray diffraction.

### Experimental

Formic acid (1 ml) was added to a stirred solution of acenaphthenequinone (0.25 g, 1.37 mmol) and 4-phenyl-2,6-dimehylaniline (0.54 g, 2.74 mmol) in methanol (20 ml). The mixture was refluxed for 24 h, then cooled and the precipitate was separated by filtration. The solid was recrystallized from ethanol/dichloromethane (*v*/*v* = 8:1), washed and dried under vacuum. Yield: 0.55 g (74%). Anal. Calcd. for C_40_H_32_N_2_: C, 88.85; H, 5.97; N, 5.18. Found: C, 88.91; H, 6.03; N, 5.06. Crystals suitable for X-ray structure determination were grown from a solution of the title compound in a mixture of cyclohexane/dichloromethane (1:2, *v*/*v*) by slow evaporation.

### Refinement

All hydrogen atoms were placed in calculated positions with C—H distances of 0.93 and 0.96 Å for aryl and methyl type H-atoms, respectively. They were included in the refinement in a riding model approximation. The H-atoms were assigned *U*iso = 1.2 times *U*_eq_ of the aryl C atoms and 1.5 times *U*_eq_ of the methyl C atoms.

### Crystal data

**Table:**

C_40_H_32_N_2_	*F*(000) = 1144
*M**_r_* = 540.68	*D*_x_ = 1.165 Mg m^−^^3^
Monoclinic, *C*2/*c*	Mo *K*α radiation, λ = 0.71073 Å
*a* = 22.994 (14) Å	Cell parameters from 3073 reflections
*b* = 8.676 (5) Å	θ = 2.1–25.7°
*c* = 18.652 (18) Å	µ = 0.07 mm^−^^1^
β = 124.084 (4)°	*T* = 296 K
*V* = 3082 (4) Å^3^	Block, red
*Z* = 4	0.23 × 0.21 × 0.19 mm

### Data collection

**Table:**

Bruker APEXII CCD diffractometer	2857 independent reflections
Radiation source: fine-focus sealed tube	1596 reflections with *I* > 2σ(*I*)
graphite	*R*_int_ = 0.031
φ and ω scans	θ_max_ = 25.5°, θ_min_ = 2.1°
Absorption correction: multi-scan (*SADABS*; Sheldrick, 2008*a*)	*h* = −27→27
*T*_min_ = 0.985, *T*_max_ = 0.987	*k* = −10→10
10667 measured reflections	*l* = −22→20

### Refinement

**Table:**

Refinement on *F*^2^	Primary atom site location: structure-invariant direct methods
Least-squares matrix: full	Secondary atom site location: difference Fourier map
*R*[*F*^2^ > 2σ(*F*^2^)] = 0.059	Hydrogen site location: inferred from neighbouring sites
*wR*(*F*^2^) = 0.211	H-atom parameters constrained
*S* = 1.16	*w* = 1/[σ^2^(*F*_o_^2^) + (0.0705*P*)^2^ + 3.4006*P*] where *P* = (*F*_o_^2^ + 2*F*_c_^2^)/3
2857 reflections	(Δ/σ)_max_ < 0.001
193 parameters	Δρ_max_ = 0.23 e Å^−^^3^
0 restraints	Δρ_min_ = −0.20 e Å^−^^3^

### Special details

**Table:**

Geometry. All e.s.d.'s (except the e.s.d. in the dihedral angle between two l.s. planes) are estimated using the full covariance matrix. The cell e.s.d.'s are taken into account individually in the estimation of e.s.d.'s in distances, angles and torsion angles; correlations between e.s.d.'s in cell parameters are only used when they are defined by crystal symmetry. An approximate (isotropic) treatment of cell e.s.d.'s is used for estimating e.s.d.'s involving l.s. planes.
Refinement. Refinement of *F*^2^ against ALL reflections. The weighted *R*-factor *wR* and goodness of fit *S* are based on *F*^2^, conventional *R*-factors *R* are based on *F*, with *F* set to zero for negative *F*^2^. The threshold expression of *F*^2^ > σ(*F*^2^) is used only for calculating *R*-factors(gt) *etc*. and is not relevant to the choice of reflections for refinement. *R*-factors based on *F*^2^ are statistically about twice as large as those based on *F*, and *R*- factors based on ALL data will be even larger.

### Fractional atomic coordinates and isotropic or equivalent isotropic displacement parameters (Å^2^)

**Table:**

	*x*	*y*	*z*	*U*_iso_*/*U*_eq_	
C1	0.2760 (2)	0.8437 (6)	0.7549 (2)	0.1024 (15)	
H1	0.2411	0.7699	0.7368	0.123*	
C2	0.3295 (3)	0.8543 (8)	0.8419 (3)	0.132 (2)	
H2	0.3303	0.7877	0.8815	0.159*	
C3	0.3807 (3)	0.9614 (9)	0.8696 (3)	0.135 (3)	
H3	0.4158	0.9705	0.9283	0.162*	
C4	0.3807 (2)	1.0559 (8)	0.8112 (4)	0.126 (2)	
H4	0.4165	1.1278	0.8300	0.151*	
C5	0.3271 (2)	1.0452 (6)	0.7229 (3)	0.1089 (16)	
H5	0.3277	1.1089	0.6832	0.131*	
C6	0.27359 (18)	0.9396 (5)	0.6951 (2)	0.0804 (11)	
C7	0.21565 (18)	0.9254 (4)	0.6012 (2)	0.0691 (9)	
C8	0.22787 (19)	0.9461 (4)	0.5373 (2)	0.0719 (10)	
H8	0.2729	0.9726	0.5534	0.086*	
C9	0.1758 (2)	0.9289 (4)	0.4504 (2)	0.0707 (10)	
C10	0.10860 (19)	0.8859 (3)	0.42739 (19)	0.0639 (9)	
C11	0.09402 (17)	0.8687 (4)	0.4894 (2)	0.0668 (9)	
C12	0.14776 (18)	0.8885 (4)	0.5757 (2)	0.0720 (10)	
H12	0.1382	0.8768	0.6177	0.086*	
C13	0.02861 (17)	0.7530 (3)	0.29823 (18)	0.0617 (9)	
C14	0.04351 (15)	0.5886 (3)	0.32523 (18)	0.0526 (8)	
C15	0.0000	0.5015 (4)	0.2500	0.0497 (10)	
C16	0.0000	0.3396 (5)	0.2500	0.0642 (12)	
C17	0.0457 (2)	0.2681 (4)	0.3300 (2)	0.0833 (12)	
H17	0.0476	0.1610	0.3334	0.100*	
C18	0.08782 (19)	0.3537 (4)	0.4034 (2)	0.0753 (10)	
H18	0.1177	0.3028	0.4555	0.090*	
C19	0.08738 (16)	0.5154 (3)	0.4025 (2)	0.0619 (9)	
H19	0.1162	0.5712	0.4532	0.074*	
C20	0.1907 (3)	0.9524 (5)	0.3822 (2)	0.1042 (15)	
H20A	0.2376	0.9908	0.4088	0.156*	
H20B	0.1579	1.0253	0.3402	0.156*	
H20C	0.1861	0.8559	0.3542	0.156*	
C21	0.02117 (19)	0.8275 (5)	0.4649 (3)	0.0984 (14)	
H21A	−0.0123	0.8974	0.4212	0.148*	
H21B	0.0194	0.8350	0.5151	0.148*	
H21C	0.0101	0.7240	0.4430	0.148*	
N1	0.05454 (16)	0.8787 (3)	0.33777 (16)	0.0755 (9)	

### Atomic displacement parameters (Å^2^)

**Table:**

	*U*^11^	*U*^22^	*U*^33^	*U*^12^	*U*^13^	*U*^23^
C1	0.081 (3)	0.157 (4)	0.056 (2)	0.001 (3)	0.030 (2)	−0.013 (3)
C2	0.095 (3)	0.217 (7)	0.060 (3)	0.012 (4)	0.028 (3)	−0.017 (3)
C3	0.071 (3)	0.239 (7)	0.071 (3)	0.023 (4)	0.025 (3)	−0.055 (4)
C4	0.070 (3)	0.205 (6)	0.098 (4)	−0.027 (3)	0.044 (3)	−0.082 (4)
C5	0.080 (3)	0.158 (5)	0.082 (3)	−0.031 (3)	0.041 (2)	−0.059 (3)
C6	0.064 (2)	0.113 (3)	0.060 (2)	−0.005 (2)	0.0321 (19)	−0.030 (2)
C7	0.070 (2)	0.074 (2)	0.054 (2)	−0.0068 (17)	0.0291 (18)	−0.0192 (17)
C8	0.077 (2)	0.070 (2)	0.063 (2)	−0.0172 (18)	0.0356 (19)	−0.0174 (17)
C9	0.095 (3)	0.0485 (18)	0.058 (2)	−0.0117 (18)	0.036 (2)	−0.0076 (15)
C10	0.084 (2)	0.0305 (15)	0.0465 (19)	0.0000 (15)	0.0174 (18)	−0.0051 (12)
C11	0.063 (2)	0.0528 (19)	0.060 (2)	−0.0019 (15)	0.0198 (17)	−0.0129 (15)
C12	0.067 (2)	0.082 (2)	0.059 (2)	−0.0036 (18)	0.0293 (18)	−0.0152 (17)
C13	0.080 (2)	0.0283 (15)	0.0504 (17)	−0.0004 (14)	0.0208 (16)	−0.0002 (12)
C14	0.0646 (18)	0.0322 (15)	0.0498 (17)	0.0021 (13)	0.0252 (15)	0.0007 (12)
C15	0.063 (2)	0.0282 (19)	0.051 (2)	0.000	0.027 (2)	0.000
C16	0.078 (3)	0.036 (2)	0.064 (3)	0.000	0.031 (3)	0.000
C17	0.102 (3)	0.0344 (17)	0.087 (3)	0.0073 (17)	0.036 (2)	0.0108 (17)
C18	0.086 (2)	0.050 (2)	0.065 (2)	0.0111 (17)	0.0268 (19)	0.0197 (17)
C19	0.073 (2)	0.0420 (17)	0.0547 (19)	0.0052 (15)	0.0257 (17)	0.0054 (14)
C20	0.145 (4)	0.091 (3)	0.073 (3)	−0.020 (3)	0.059 (3)	0.001 (2)
C21	0.067 (2)	0.105 (3)	0.091 (3)	−0.003 (2)	0.025 (2)	−0.028 (2)
N1	0.100 (2)	0.0295 (13)	0.0510 (16)	−0.0021 (13)	0.0140 (15)	−0.0045 (11)

### Geometric parameters (Å, °)

**Table:**

C1—C6	1.368 (6)	C12—H12	0.9300
C1—C2	1.386 (6)	C13—N1	1.263 (4)
C1—H1	0.9300	C13—C14	1.487 (4)
C2—C3	1.355 (8)	C13—C13^i^	1.522 (6)
C2—H2	0.9300	C14—C19	1.368 (4)
C3—C4	1.363 (8)	C14—C15	1.403 (3)
C3—H3	0.9300	C15—C14^i^	1.403 (3)
C4—C5	1.402 (6)	C15—C16	1.404 (6)
C4—H4	0.9300	C16—C17^i^	1.400 (4)
C5—C6	1.381 (6)	C16—C17	1.400 (4)
C5—H5	0.9300	C17—C18	1.370 (5)
C6—C7	1.497 (5)	C17—H17	0.9300
C7—C8	1.381 (5)	C18—C19	1.403 (4)
C7—C12	1.391 (5)	C18—H18	0.9300
C8—C9	1.382 (4)	C19—H19	0.9300
C8—H8	0.9300	C20—H20A	0.9600
C9—C10	1.404 (5)	C20—H20B	0.9600
C9—C20	1.504 (5)	C20—H20C	0.9600
C10—C11	1.381 (5)	C21—H21A	0.9600
C10—N1	1.419 (4)	C21—H21B	0.9600
C11—C12	1.385 (4)	C21—H21C	0.9600
C11—C21	1.512 (5)		
			
C6—C1—C2	121.2 (5)	C7—C12—H12	119.2
C6—C1—H1	119.4	N1—C13—C14	133.3 (3)
C2—C1—H1	119.4	N1—C13—C13^i^	120.18 (17)
C3—C2—C1	120.3 (6)	C14—C13—C13^i^	106.44 (15)
C3—C2—H2	119.9	C19—C14—C15	119.7 (3)
C1—C2—H2	119.9	C19—C14—C13	134.2 (3)
C2—C3—C4	119.8 (5)	C15—C14—C13	106.2 (2)
C2—C3—H3	120.1	C14^i^—C15—C14	114.8 (3)
C4—C3—H3	120.1	C14^i^—C15—C16	122.62 (17)
C3—C4—C5	120.4 (5)	C14—C15—C16	122.62 (17)
C3—C4—H4	119.8	C17^i^—C16—C17	127.3 (4)
C5—C4—H4	119.8	C17^i^—C16—C15	116.3 (2)
C6—C5—C4	119.8 (5)	C17—C16—C15	116.3 (2)
C6—C5—H5	120.1	C18—C17—C16	120.8 (3)
C4—C5—H5	120.1	C18—C17—H17	119.6
C1—C6—C5	118.5 (4)	C16—C17—H17	119.6
C1—C6—C7	120.4 (4)	C17—C18—C19	122.3 (3)
C5—C6—C7	121.1 (4)	C17—C18—H18	118.8
C8—C7—C12	117.9 (3)	C19—C18—H18	118.8
C8—C7—C6	121.4 (3)	C14—C19—C18	118.2 (3)
C12—C7—C6	120.8 (3)	C14—C19—H19	120.9
C9—C8—C7	122.5 (3)	C18—C19—H19	120.9
C9—C8—H8	118.7	C9—C20—H20A	109.5
C7—C8—H8	118.7	C9—C20—H20B	109.5
C8—C9—C10	117.9 (3)	H20A—C20—H20B	109.5
C8—C9—C20	121.3 (4)	C9—C20—H20C	109.5
C10—C9—C20	120.8 (3)	H20A—C20—H20C	109.5
C11—C10—C9	121.0 (3)	H20B—C20—H20C	109.5
C11—C10—N1	121.2 (3)	C11—C21—H21A	109.5
C9—C10—N1	117.3 (3)	C11—C21—H21B	109.5
C10—C11—C12	118.9 (3)	H21A—C21—H21B	109.5
C10—C11—C21	121.4 (3)	C11—C21—H21C	109.5
C12—C11—C21	119.7 (4)	H21A—C21—H21C	109.5
C11—C12—C7	121.7 (3)	H21B—C21—H21C	109.5
C11—C12—H12	119.2	C13—N1—C10	122.7 (2)
			
C6—C1—C2—C3	0.3 (8)	C21—C11—C12—C7	179.6 (3)
C1—C2—C3—C4	−2.0 (8)	C8—C7—C12—C11	1.7 (5)
C2—C3—C4—C5	1.5 (8)	C6—C7—C12—C11	−177.3 (3)
C3—C4—C5—C6	0.8 (7)	N1—C13—C14—C19	4.2 (7)
C2—C1—C6—C5	2.0 (7)	C13^i^—C13—C14—C19	−178.1 (4)
C2—C1—C6—C7	179.5 (4)	N1—C13—C14—C15	−176.1 (4)
C4—C5—C6—C1	−2.5 (6)	C13^i^—C13—C14—C15	1.6 (4)
C4—C5—C6—C7	180.0 (4)	C19—C14—C15—C14^i^	179.1 (3)
C1—C6—C7—C8	−142.6 (4)	C13—C14—C15—C14^i^	−0.65 (17)
C5—C6—C7—C8	34.9 (5)	C19—C14—C15—C16	−0.9 (3)
C1—C6—C7—C12	36.4 (5)	C13—C14—C15—C16	179.35 (17)
C5—C6—C7—C12	−146.1 (4)	C14^i^—C15—C16—C17^i^	0.5 (2)
C12—C7—C8—C9	−1.1 (5)	C14—C15—C16—C17^i^	−179.5 (2)
C6—C7—C8—C9	177.9 (3)	C14^i^—C15—C16—C17	−179.5 (2)
C7—C8—C9—C10	−1.3 (5)	C14—C15—C16—C17	0.5 (2)
C7—C8—C9—C20	179.9 (3)	C17^i^—C16—C17—C18	179.8 (4)
C8—C9—C10—C11	3.2 (5)	C15—C16—C17—C18	−0.2 (4)
C20—C9—C10—C11	−178.0 (3)	C16—C17—C18—C19	0.1 (6)
C8—C9—C10—N1	175.9 (3)	C15—C14—C19—C18	0.8 (5)
C20—C9—C10—N1	−5.3 (5)	C13—C14—C19—C18	−179.5 (4)
C9—C10—C11—C12	−2.7 (5)	C17—C18—C19—C14	−0.5 (6)
N1—C10—C11—C12	−175.1 (3)	C14—C13—N1—C10	−1.7 (7)
C9—C10—C11—C21	177.9 (3)	C13^i^—C13—N1—C10	−179.2 (4)
N1—C10—C11—C21	5.5 (5)	C11—C10—N1—C13	−78.7 (5)
C10—C11—C12—C7	0.2 (5)	C9—C10—N1—C13	108.6 (4)

Symmetry codes: (i) −*x*, *y*, −*z*+1/2.
